# The Cost of Provisions

**Published:** 1905-10-14

**Authors:** 


### EDITOR'S LETTER-BOX.

Our Correspondents are reminded that prolixity is a great bar to publication, and that brevity of style and conciseness of statement greatly facilitate early insertion.]

### THE COST OF PROVISIONS.

A Matron writes:—I am matron of a small surgical hospital with accommodation for fifteen patients. Our staff consists of an assistant nurse, two probationers, a porter, and two servants, beside myself. Each member of the staff is a total abstainer from principle. As a rule the patients have good appetites. A bad accident or operation case does not depress them for any length of time. I was recently working out the average cost per person per day for the year ending June 30, 1905. For articles of diet alone the average individual cost per day in each month ranges from 8½d. to 9¾d., and the daily average throughout the year is 9½d. We can accommodate fifteen patients. Our average for the past year was 11.6 beds occupied per day. No difference is made in the provision of food, with the exception of occasionally a little extra for the staff for breakfast and supper.

